# Delivery of mental health treatment to combat veterans with psychiatric diagnoses and TBI histories

**DOI:** 10.1371/journal.pone.0184265

**Published:** 2017-09-08

**Authors:** Shannon R. Miles, Juliette M. Harik, Natalie E. Hundt, Joseph Mignogna, Nicholas J. Pastorek, Karin E. Thompson, Jessica S. Freshour, Hong J. Yu, Jeffrey A. Cully

**Affiliations:** 1 Health Service Research and Development Center of Innovation on Disability and Rehabilitation Research (CINDRR), James A. Haley Veterans’ Hospital, Tampa, Florida, United States of America; 2 Department of Psychiatry & Behavioral Neurosciences, Morsani College of Medicine, University of South Florida, Tampa, Florida; 3 National Center for PTSD-Executive Division, VA Medical Center (116D), White River Junction, Vermont, United States of America; 4 Geisel School of Medicine at Dartmouth, Hanover, New Hampshire, United States of America; 5 VA South Central Mental Illness Research, Education, and Clinical Center, Michael E DeBakey VA Medical Center (MEDVAMC 152), Houston, Texas, United States of America; 6 Baylor College of Medicine, One Baylor Plaza, Houston, Texas, United States of America; 7 VA HSR&D Houston Center for Innovations in Quality, Effectiveness and Safety, MEDVAMC (152), Houston, Texas, United States of America; 8 Center of Excellence for Research on Returning War Veterans, Waco, Texas, United States of America; 9 Central Texas Veterans Healthcare System, Waco, TX Texas A&M Health Science Center, Temple, Texas, United States of America; 10 Texas A&M Health Science Center, Temple, Texas, United States of America; 11 Rehabilitation and Extended Care Line, MEDVAMC, Houston, Texas, United States of America; Hunter Holmes McGuire VA Medical Center, UNITED STATES

## Abstract

Traumatic brain injury (TBI) and mental health (MH) disorders are prevalent in combat veterans returning from Afghanistan and/or Iraq (hereafter referred to as returning veterans). Accurate estimates of service utilization for veterans with and without TBI exposure (referred to as TBI history) are imperative in order to provide high quality healthcare to returning veterans. We examined associations between TBI history and MH service utilization in a subsample of returning veterans who were newly diagnosed with posttraumatic stress disorder (PTSD), depression, and/or anxiety in the 2010 fiscal year (N = 55,458). Data were extracted from the Veterans Health Administration (VHA) National Patient Care Database. Veterans with MH diagnoses and TBI histories attended significantly more psychotherapy visits, (*M* = 8.32 visits, *SD* = 17.15) and were more likely to attend at least 8 psychotherapy visits, (15.7%) than veterans with MH diagnoses but no TBI history (*M* = 6.48 visits, *SD* = 12.12; 10.1% attended at least 8 sessions). PTSD and TBI history, but not depression or anxiety, were associated with a greater number of psychotherapy visits when controlling for demographic and clinical variables. PTSD, anxiety, depression, and TBI history were associated with number of psychotropic medication-management visits. TBI history was related to greater MH service utilization, independent of MH diagnoses. Future research should examine what MH services are being utilized and if these services are helping veterans recover from their disorders.

## Introduction

Since 2001, over 2.6 million Operation Enduring Freedom (OEF), Operation Iraqi Freedom (OIF), and Operation New Dawn (OND) veterans (hereafter referred to as returning veterans) have left active duty and become eligible for health care through the Veterans Health Administration (VHA) [1]. Prevalent mental health (MH) disorders (as defined by the International Classification of Diseases–9th edition) for veterans receiving VHA care include posttraumatic stress disorder (PTSD), depressive disorders, and anxiety disorders. These disorders can impair interpersonal, psychological, and occupational functioning [2,3] and are a top VHA treatment priority [1].

Traumatic brain injury (TBI) is another common condition among veterans, given that more than 300,000 service members have experienced a TBI since 2001 [4]. TBIs of mild severity (mTBI) are most common and are characterized by a temporary change in mental status, followed by possible cognitive (e.g., difficulty remembering or concentrating), physical (e.g., headaches, nausea, fatigue, sleep difficulties, sensitivity to light or sounds), and emotional (e.g., irritability, anxiety, depression) symptoms that usually remit within 3 to 12 months [5]. Yet, a study of service members deployed to Iraq and/or Afghanistan found that 35% of those who experienced an in-theater mTBI reported three or more current neuropsychiatric symptoms following a possible mTBI, five or more months after their most recent deployment [6].

Moderate and severe TBI are less common but can result in significant activity limitations and participation restrictions [7]. To address the needs of returning veterans, the VHA established a nationwide screening process to assess veterans for TBI exposure (called TBI history) and current symptoms that may or may not be related to the TBI [8]. By 2008, almost 92% of veterans had received the screen; 25% of those had screened positive for TBI history [9]. Yet, self-report symptoms after an mTBI are non-specific to the head injury and are found in samples with other injuries [10].

Psychiatric symptoms such as those needed to diagnosis PTSD, depression, and anxiety are related to self-reported TBI symptoms [10]. PTSD and TBI can independently impact health status and are frequently comorbid. They are the signature injuries of OEF/OIF, and about 20% of those with TBI histories also have PTSD [11,12]. Service members who experienced higher levels of combat stress reported more symptoms after an mTBI than those who experienced low combat stress [13]. Comorbid PTSD and mTBI have been related to increased PTSD symptom severity [14,15] and greater healthcare costs [16]. However, PTSD is not the only psychiatric comorbidity associated with TBI history.

Although less studied in veteran samples, depression and other anxiety disorders are also commonly comorbid among those with TBI histories. There is wide variability in the estimates of psychiatric diagnoses post-TBI because of the diverse samples studied, including epidemiological, inpatient, and outpatient. With this caveat in mind, between 23% and 38% of civilians with mTBI histories are diagnosed with an anxiety disorder [17,18] within five years post head injury. Between 14% and 61% of people who sustained a TBI developed depression [19,20] within one [19] to eight years [20] postinjury, making depression one of the most common psychiatric comorbidities of mTBI [21]. The association between TBI and depression extends to service-member populations [22]; however, the focus on PTSD and TBI has overshadowed it. Individuals who develop an anxiety or depressive disorder after TBI are more functionally impaired [23] and are more likely to have a complicated and longer recovery than those with TBI histories and no psychiatric disorder [24]. To further confound the research, anxiety, depression, and PTSD symptoms, including subjective feelings of nervousness, sleep difficulties, fatigue, and attentional impairments, overlap with one another and with TBI [25].

Veterans with TBI histories may incorrectly attribute their symptoms to the head injury rather than a MH disorder or psychiatric distress. Recently, lay and medical attention has focused on the long-term effects of TBI, which may increase the concern veterans feel when they are diagnosed with a TBI history. However, accumulating evidence indicates that long-term mTBI symptoms (>12 months) are primarily related to psychological factors, such as premorbid or concurrent MH diagnoses, rather than solely to the original head injury [10, 26–28]. Another reason why veterans may continue to report long-term TBI symptoms is that the veterans may be working under the reasonable assumption that demonstrating impairment and/or disability through the utilization of health care services will provide support for disability claims that may be under review at the Veterans Benefits Administration [29].

If long term mTBI symptoms are primarily related to psychological factors, then MH service use may not be affected by TBI history because psychological symptoms may already be the focus of treatment for veterans identified as having a psychiatric disorder. Conversely, service utilization may be affected by TBI histories, as veterans with TBI histories can be more clinically complex with greater severity of psychological symptoms [14,15]. Consistent with both of these possibilities, previous research results are equivocal in terms of the number and types of services used by veterans with TBI histories. A large study found that OEF/OIF veterans (N = 1,746) with mTBI histories used more MH, primary care, and emergency services than veterans without mTBI histories, even after controlling for MH screen results [30]. Contrasting results in smaller samples showed veterans with mTBI histories did not differ from those without mTBI histories in terms of use of psychiatric medication [31], referrals to substance-abuse treatment [32], pain medication [33], receipt of at least one MH counseling visit [31], and number of Cognitive Processing Therapy [15] or Prolonged Exposure Therapy sessions [34].

It is imperative to understand the service utilization patterns of this younger generation of veterans as their war experiences have differed from previous generations. Younger veterans are more likely to serve in multiple deployments of greater durations, have less time between deployments, and return to a bombardment of information about MH disorders and TBI sequelae [12]. Although studies examining the impact of TBI history on service utilization among PTSD samples have yielded equivocal results, there is simply a lack of research examining how TBI history relates to service utilization in veterans with anxiety and depressive disorders. This study advances the literature by examining how comorbid TBI is associated with MH service utilization in a national subsample of returning veterans newly diagnosed with PTSD, depression, and/or anxiety. Lingering TBI symptoms [26–28] may be related to psychological factors and not the head injury, thus in a sample diagnosed with psychiatric disorders, veterans with TBI histories may not differ from veterans without TBI histories in their MH service utilization. This research clarifies how returning veterans with MH disorders and comorbid TBI histories use VHA MH services.

## Methods

### Participants

Participants included a subset of OEF/OIF veterans who received at least one new mental health (MH) index diagnosis of depression, anxiety disorder, or PTSD in the VHA in the 2010 fiscal year (October 1, 2009 to September 30, 2010). We restricted the sample to OEF/OIF veterans, including only those born in 1973 or later (modeled after Brooks et al., [35] and Rosenheck & Fontana, [36]), which allowed us to remove veterans who served in earlier conflicts. Because combat veterans have the highest rates of TBI histories due to war injuries and have been inundated with information about TBI, we restricted the sample to combat veterans, using the combat-veteran eligibility-indicator variable that specifies whether a veteran served in active duty in a theater of combat operations during a period of war after the Gulf War or after November 1998 (VA Information Resource Center [VIReC] [37]). According to the VIReC website, by 2010 no veteran was missing the combat- indicator variable [38]. Finally, we included veterans who had the opportunity to use outpatient services by excluding veterans who had 60 or more inpatient hospital days in the 180 days following the index diagnosis date (defined below), as these veterans were more likely to receive inpatient services, which we did not measure. Additionally, we wanted all veterans in this study to have similar follow up periods, and veterans who were hospitalized for extended time frames would not have the same follow up period as veterans who were not hospitalized.

We defined new “index” diagnosis as the presence of a new anxiety, depression, and/or PTSD diagnosis without any MH diagnoses in the six months (clean period) prior to the index date. The veterans could have had a MH diagnosis before the six month clean period or a diagnosis given by a non-VHA provider. The veteran’s symptoms likely began before the index date; however, the index date marks the first time veterans would have access to VHA MH services since each service encounter identifies the problem(s) treated during that visit. In order to capture the most comprehensive sample of veterans with psychiatric disorders, MH diagnoses were not restricted to MH clinics and could have been given by any VHA provider. We used the following International Classification of Diseases–9^th^ edition—Clinician Modification (ICD-9-CM) codes to determine MH diagnoses of the most common mental health problems for returning veterans: depression, including major depressive disorder, dysthymia, depression not otherwise specified, and mood disorder due to a general medical condition (293.83, 296.20–296.36, 300.4, 311); anxiety disorders (293.84, 293.89, 300.00–300.02, 300.09, 300.20–300.23, 300.29, 300.3); and PTSD and/or acute stress disorder (308, 309.81). Veterans could have more than one MH diagnosis.

### Procedures

A retrospective database study was conducted to examine MH diagnoses and MH service use for returning veterans with and without TBI histories. The VHA maintains data extracted from veterans’ electronic medical records in the VHA National Patient Care Database (NPCD). It scrambles Social Security numbers to protect veterans’ identities while still allowing patient and service characteristics to be studied. The accuracy and validity of the NPCD data are monitored by the VHA VIReC [37]. Veterans in the current study were already enrolled in the VHA system; we examined MH service utilization data from the date of the index MH diagnosis to one year after this date. Demographic variables were extracted on the same date as the index diagnosis. Psychiatric comorbidities were extract from the index date to one year post index date, the same time frame as the service utilization. TBI diagnoses were extracted from 2001 (beginning of OEF) to six months after the index diagnosis. TBI could have occurred during service related duties or civilian activities as the VHA provides care to veterans with TBI regardless of where the injury is sustained. TBI history and psychiatric comorbidities were included if any VHA encounter listed them during the respective timeframes. The study was approved by the Baylor College of Medicine Institutional Review Board and Houston VA Research and Development Committee prior to data examination. The study conforms to all American Psychological Association ethical guidelines and to all federal research laws.

### Variables of interest

#### Demographics

Demographics included age (continuous variable), sex (man or woman), ethnicity (mutually exclusive categorical variable), marital status (married, not married), service-connected disability percentage (categorized into 0%, 1–49%, or 50%+), distance to VHA in miles (continuous variable), and MH diagnoses (either present or absent).

#### Mental health service utilization

We used Current Procedural Terminology (CPT) codes to the veteran’s use of outpatient MH services, including individual psychotherapy (90804, 90806, 90808, 90810, 90812, 90814, 90845, 90875, 90876, 96152), group therapy (90849, 90853, 90857, 96153), family therapy (90846, 90847, 96154, 96155), and medication-management visits (90805, 90807, 90809, 90811, 90813, 90815, 90862).

#### Traumatic brain injury

Diagnoses require a provider to give an actual diagnosis rather than rely on a screen that is non-specific, thus we selected TBI ICD-9 codes rather than TBI screen results. ICD-9 codes do not reliably distinguish between those with mTBI and those with moderate and severe TBI [39], so all ICD-9 TBI codes were included (310.2, 800.xx-801.xx, 803.xx-804.xx, 850.xx-854.xx, 905.0, 907.0, 950.1-.3, 959.01, 959.9, V15.52). However, between 81% and 83% [4,39] of veterans with TBI have sustained a TBI of mild severity, making it likely that most of our sample identified as having a TBI history had experienced an mTBI. Within our sample of veterans with a new onset MH diagnosis, 308 veterans were diagnosed with TBI histories from 2001–2006, and the following numbers were diagnosed in the following time periods; 708 in 2007; 2,036 in 2008; 1,824 in 2009; 5,996 in 2010; and 479 in 2011. We were interested in TBI histories with comorbid MH diagnoses; thus, there were no veterans who had TBI histories and lacked psychiatric comorbidities.

#### Mental health comorbidities

To examine the potential effect of MH comorbidities (other than the index diagnosis), we examined substance-use disorders (ICD-9 codes: 303 [.9], 304.0-.6, [.9], 305, 305.2-.7, 305.9), psychotic disorders, (293.81- .82, 293.89, 298.8 - .9, 295 [.4, .9, .7], 297.1, 298 [.8]), bipolar disorders (296.40 -.46, 296.50–56, 296.8), and personality disorders (301 [.0, .2, .22, .4, .5, .6, .7, .81, .82, .83, .89, .9]) in any VHA encounter in the year following the index diagnosis.

### Data analysis

Data analyses were conducted using SAS version 9.2 (SAS Institute, Inc., Cary, NC). Independent samples *t*-tests examined whether veterans with and without TBI histories differed in number of therapy and medication visits, using adjusted degrees of freedom to account for unequal variances. A chi-square was used to detect differences in the proportion of veterans with and without TBI histories who completed eight or more psychotherapy visits, which was modeled after previous studies to identify if the veteran received the minimum number of sessions associated with an adequate dose of sessions for evidence-based psychotherapies (EBP) for PTSD, depression, and anxiety [40,41]. While the content and quality of the treatment sessions were unknow, any veteran receiving fewer than eight sessions did not have the opportunity to complete a full EBP [40,41,42]. Two multiple regression models predicted number of psychotherapy and medication- management visits, respectively, with demographic factors, psychiatric diagnoses, and TBI history as predictors. Male sex, being married, having any service-connected disability, and the presence of each psychiatric disorder were the defaults for the categorical dummy-coded variables. Because all index diagnoses and psychiatric comorbidities were dummy coded for the multiple regression models, all diagnostic variables’ unstandardized regression coefficients were directly comparable [43]. Categorical variables with more than two levels were dummy coded to create additional variables (e.g., distance to the VHA was dummy coded into: 4–10 miles, 10–19 miles, and 20 or more miles, with the constant being 0–3 miles; Table 1). In consideration of the large sample size and Type I error, a statistical-significance level was set at alpha = .01 (based on Table 2 which has five comparisons α = .05/5 = .01), and effect sizes were provided.

**Table 1:** Demographics of OEF/OIF veterans with new MH diagnoses, with and without TBI histories (N = 55,458).

	MH only	MH and TBI			
	(*n* = 44,107)	(*n* = 11,351)	*F or X*^*2*^	*p* value	*Effect size*
Mean age (SD)	28.18 (3.76)	27.67 (3.68)	*F* = 162.22	< .0001	*d* = .*137*
Male sex (%)	84.85	94.64	*X*^*2*^ = 903.19	< .0001	***φ*** = .128
Ethnicity (%)			*X*^*2*^ = 236.47	< .0001	V = .065
Black	13.87	8.91			
White	69.52	74.80			
Others	4.88	5.47			
Unknown	11.74	10.83			
Marital status (%)			*X*^*2*^ = 17.78	.0001	V = .018
Married	38.04	40.10			
Not married	59.08	57.33			
Unknown	2.87	2.56			
Average distance to VHA in miles (SD)	12.83 (14.83)	13.11 (14.04)	F = 3.25	.0716	*d* = .*019*
Income					
Income (%)			*X*^*2*^ = 46.95	< .0001	V = .017
$0	1.42	1.37			
$1 = $30,000	5.52	4.34			
$30,000–39,999	24.73	23.50			
$40,000–49,999	30.80	30.69			
$50,000–59,999	17.43	18.13			
> = $60,000	20.10	21.95			
Service-connected category (%)			*X*^*2*^ = 732.20	< .0001	V = .115
0	53.59	44.79			
1–49%	25.62	22.02			
50%+	20.79	33.19			
Comorbid psychiatric diagnoses (% with disorder)					
Bipolar	1.26	1.04	*X*^*2*^ = 3.67	.0601	***φ*** = .008
Psychotic	1.10	1.29	*X*^*2*^ = 2.71	.0100	***φ*** = .007
Substance	33.41	38.80	*X*^*2*^ = 116.19	< .0001	***φ*** = .002
Personality disorder	1.51	1.51	*X*^*2*^ = .0007	.9783	***φ*** = .000

OEF = Operation Enduring Freedom; OIF = Operation Iraqi Freedom; MH = mental health; TBI = traumatic brain injury; VHA = Veterans Health Administration

**Table 2:** Psychotherapy and medication visits at 12-months follow-up (*N* = 55,458).

	Any therapy	Individual therapy	Group therapy	Family therapy	Medication visits
**Entire cohort N (%)**	25,548 (46.07)	24,028 (43.33)	5,522 (9.96)	1,249 (2.25)	26,959 (48.61)
Mean no. of visits (SD)	6.91 (13.50)	4.80 (6.28)	10.33 (21.23)	3.29 (5.02)	3.24 (3.41)
Median	3	3	4	2	2
Mode	1	1	1	1	1
**MH group (n)**	19,522	18,357	4,021	903	20,843
Mean no. of visits (SD)	6.48 (12.12)	4.58 (6.07)	9.79 (18.81)	3.34 (4.95)	3.18 (3.29)
Median	3	2	4	1	2
Mode	1	1	4	1	1
**MH + TBI group (n)**	6,026	5,671	1,501	346	6,116
Mean no. of visits (SD)	8.32 (17.15)	5.53 (6.88)	11.76 (26.61)	3.18 (5.14)	3.46 (3.79)
Median	4	3	4	2	2
Mode	1	1	1	1	2
**Difference between no. of visits for MH and MH+TBI groups**	*t*(7966) = 7.76**	*t*(8573) = 9.39**	*t*(7493) = 4.93**	*t*(2085) = 2.63**	*t*(8978) = 5.31**
Cohen’s *d*	.11	.12	.09	.04	.08

MH = mental health. TBI = traumatic brain injury.

**p < .01.

## Results

Of the 583,733 veterans in the VHA system who received a new-onset MH diagnosis in 2010, 84,255 (14%) were born after 1973 (our OEF/OIF variable); and, of these, 55,458 (66%) had served in combat and comprised our study sample. Within this combat OEF/OIF subsample, 21,110 (38%) had a depressive disorder, 12,402 (22%) had an anxiety disorder, 31,709 (57%) had PTSD, and 11,351 (20%) had a TBI history (percentages sum to greater than 100% because of the comorbidity between the diagnoses). Table 1 displays the demographic information for veterans with TBI histories (MH+TBI) and those without TBI histories (MH). There were many significant but small differences between the MH and MH+TBI groups. For example, veterans with MH+TBI were younger, more often Caucasian, more likely to have a service-connected disability rating of 50% or higher, and more likely to have a substance-use disorder than veterans without TBI histories.

Across the study sample, 49% of veterans attended at least one medication visit, and 46% attended at least one psychotherapy visit in the year after receiving their index diagnosis (Table 2). There was overlap in services with 29% of the veterans receiving both psychotherapy and medications. Of those who received psychotherapy, individual therapy was most common, followed by group, and then family therapy. Independent-sample *t*-tests demonstrated that veterans with MH+TBI, on average, attended one more individual psychotherapy visit, two more group psychotherapy visits, and 0.28 more medication visits than veterans without TBI.

Table 3 displays the percentage of each group that attended 0 visits, 1–3 visits, 4–7 visits, 8–12 visits, and more than 25 visits after receiving their index diagnosis. Approximately 10% of the MH group and 16% of the MH+TBI history group attended eight or more psychotherapy visits, a statistically significant discrepancy, *X*^2^ (6, *N* = 225) = 13.93, *df* = 2, *p* < .001, ***φ*** = .25. Average time from index diagnosis to the next MH psychotherapy or medication visit was 73.96 days (*SD* = 124.60, *mdn* = 20). Veterans with MH (*M* = 74.98, *SD* = 126.49, *mdn* = 19) had a slightly longer but statistically significant time difference from the index diagnosis to next psychotherapy or medication visit than veterans with MH+TBI (*M* = 70.39, *SD* = 117.71, *mdn* = 21; *t*(15E3) = -3.13, *p* = .002, *d* = .04).

**Table 3:** Psychotherapy exposure.

	Full cohort (N = 55,458)		MH group (n = 44,107)		MH + TBI group (n = 11,351)	
	n	%	n	%	n	%
0 Visits (no therapy)	29,910	53.93	24,585	55.74	5,325	46.91
1–3 visits	13,960	25.17	10,996	24.93	2,964	26.11
4–7 visits	5,363	9.67	4,083	9.26	1,280	11.28
8–12 visits	2,759	4.97	2,024	4.59	735	6.48
12–25 visits	2,282	4.11	1,619	3.67	663	5.84
>25 visits	1,184	2.13	800	1.81	384	3.38

MH = mental health. TBI = traumatic brain injury.

Chi-square to test whether MH and MH+TBI groups differ in those who have 8 or more visits. *X*^2^ (6, *N* = 225) = 13.93, *df* = 2, *p* < .001, ***φ*** = .25.

The regression model predicting number of psychotherapy visits with demographic variables, index diagnoses, psychiatric comorbidities, and TBI history as independent variables was significant, *F*(20, 55437) = 90.05, *p* < .0001; Table 4. Several demographic and psychiatric variables were statistically associated with more psychotherapy visits, including increasing age, female sex, being married, living closer to the VHA, and not having a service-connected disability. Significant psychiatric predictors included PTSD (b = 0.80) and all comorbidities (substance use [b = 2.39], schizophrenia [b = 4.66], bipolar [b = 2.87], and personality disorders [b = 4.93]). Anxiety and depression diagnoses were not associated with number of psychotherapy visits. The index diagnoses had smaller parameter estimates than the comorbidities, likely because the sample was restricted by choosing only veterans with index diagnoses of anxiety, depression, and/or PTSD. TBI history was statistically associated with the number of psychotherapy visits (b = 1.46), even when controlling for the influence of all other predictors.

**Table 4:** Multiple regression predicting number of psychotherapy and medication visits.

	Predicting psychotherapy visits			Predicting medication visits		
	Parameter estimate	*Std*. *Error*	*t* value	Parameter estimate	*Std*. *Error*	*t* value
Intercept	1.40	.50	2.76*	0.71	.15	4.85**
Age	0.08	.01	6.74**	0.02	.00	6.28**
Male sex	-0.06	.12	-5.05**	-0.12	.04	-3.42**
Married	0.27	.09	3.08*	0.11	.03	4.38**
Income (constant = $0)						
$1 = $30,000	-1.31	.39	13.35**	-0.62	.11	-5.47**
$30,000–39,999	-1.42	.36	-3.99**	-0.62	.10	-5.98**
$40,000–49,999	-1.50	.35	-4.24**	-0.60	.10	-5.85**
$50,000–59,999	-1.61	.36	-4.48**	-0.61	.10	-5.82**
> = $60,000	-1.46	.36	-4.09**	-0.54	.10	-5.27**
Distance to VHA (constant = 0–3 miles)						
4–10 miles	0.30	.12	2.38	0.11	.04	3.28
10–19 miles	0.10	.12	0.86	0.10	.03	3.10*
> = 20 miles	-0.05	.13	-0.41	0.06	.04	1.65
Any SC	-0.86	.09	-10.19**	-0.16	.02	-6.47**
Index diagnosis						
Anxiety	0.12	.13	0.96	0.34	.04	8.89**
Depression	0.28	.11	2.53	0.48	.03	14.84**
PTSD	0.80	.12	6.58**	0.47	.04	13.33**
Comorbid MH Dx						
Substance use	2.39	.09	27.25**	0.77	.03	30.08**
Psychosis	4.66	.39	12.02**	2.22	.11	19.75**
Bipolar disorder	2.87	.38	7.64**	2.71	.11	24.82**
Personality disorder	4.93	.34	14.59**	1.68	.10	17.09**
TBI	1.46	.10	14.08**	0.34	.03	11.14**

SC = service-connected disability. PTSD = posttraumatic stress disorder. MH = mental health. Dx = diagnosis. TBI = traumatic brain injury

* = *p* < .01.

** = *p* < .001

Table 4 also displays the results of the multiple regression predicting number of medication visits, which was a statistically significant model, *F*(20, 55,437) = 158.72, *p* < .0001. Increasing age, female sex, being married, and not having a service-connected disability were significantly associated with greater number of medication visits. Having a PTSD (b = 0.47), anxiety (b = 0.34), or depression (b = 0.48) index diagnosis and all psychiatric comorbidities (substance use [b = 0.77], schizophrenia [b = 2.22], bipolar [b = 2.71], and personality disorders [b = 1.68]) were associated with having more medication visits. Finally, TBI history was significantly associated with greater number of medication visits (b = 0.34), while accounting for the influence of all other variables.

## Discussion

We examined type and quantity of MH services used by a subset of OEF/OIF combat veterans with and without TBI histories who received a new MH index diagnosis in 2010. PTSD was the most common index diagnosis, followed by depressive and then anxiety disorders. This is expected in a sample of combat veterans for whom PTSD and TBI are the “signature injuries” [1]. About 20% of the sample had a TBI history, consistent with previous estimates of OEF/OIF/OND veteran samples [1,39]. Veterans with TBI histories were more likely to be male, service connected, and have a comorbid substance use disorder diagnosis than veterans without TBI histories.

Demographic variables such as female gender, being married, and not having a service connected disability were all significantly related to greater number of medication and psychotherapy visits. Women are more likely to seek mental health treatment in general [44] and social support (such as being married) is often a motivator to seek treatment [45]. Service connected disability has a complicated relationship with number of visits, as veterans may believe that they need to have documentation of continuing symptoms in order to retain or increase their disability rating [46]. Yet, service utilization often decreases after a veteran receives a desired level of disability [47]. In our sample, having any service-connected disability was negatively associated with psychotherapy and medications visits. Future research may want to examine both the current percentage of service connected disability and if the veteran is seeking an increase in disability rating.

MH index diagnoses were also related to quantity of services. Of the three index diagnoses included (PTSD, depression, anxiety), PTSD displayed the greatest association with the number of psychotherapy visits; whereas depression had the strongest association with medication visits. This difference may be explained, at least in part, by the fact that there are a greater number of evidence-based medications available for depression than for PTSD. Additionally, VHA has developed nationwide initiatives to increase access to EBP for PTSD, thus health care resources have focused on veterans with this disorder.

All comorbid MH diagnoses were associated with more psychotherapy and medication visits. Substance use and personality disorders are generally chronic conditions that require long-term psychotherapy. If veterans presented with complex presentations that involved substances use and personality characteristics that prolong their recoveries, they used more services. MH comorbidity is common [48], and comorbidities will increase the demand for services.

Even after considering demographic characteristics and MH diagnoses, TBI history was associated with increases in MH service use. This is consistent with a previous study of OEF/OIF veterans who were screened for TBI history and MH diagnoses [30]. Maguen and colleagues’ study [30] differed from the current study in that they did not select veterans based on the presence of psychiatric diagnoses as the current study did. Other studies have found little difference in terms of MH service use for service members and veterans with and without TBI histories [31–34], however, the sample sizes of these studies were significantly smaller. Examining data from a large, national VHA cohort may provide more accurate estimates of service utilization. In the current study, the differences in service utilization for veterans with TBI histories and veterans without TBI histories equated to about three psychotherapy visits more for those with TBI histories, and across the entire VHA, this results in tens of thousands more psychotherapy visits per year.

The current data do not provide reasons why veterans with TBI histories used more MH services; however, there are possible explanations worth considering as this generation of veterans will continue to make up a larger proportion of the population served by VHA. Veterans with TBI histories may have used more services than veterans without TBI histories due to greater symptom complexity such as substance use disorders, easier access to services, an attempt to receive service connection disability, or a combination of these factors. The complexity of veterans with TBI histories [49] could be due to neurological, psychiatric, social, or other medical factors, such as the demographic and psychiatric comorbidity differences found between the veterans with and without TBI histories in this study. Another possibility is that veterans identified as having a possible TBI history gained access to specialized clinics (i.e., polytrauma clinics) that offer a variety of mental health services, such as sleep hygiene and pain and stress management. Psychologists are often included in polytrauma clinics [50]; thus, veterans with TBI histories may have access to MH providers in medical settings in addition to providers in MH clinics.

Finally, Veterans with TBI histories attended more psychotherapy visits and had shorter wait times until their first follow up visit than veterans without TBI histories. However, there remains opportunity to improve service utilization rates as only 46% of the entire sample attended at least one psychotherapy visit. This is consistent with civilian literature which suggests that 43% of diagnosed patients attend any psychotherapy visit [44], but also demonstrates the gap between those with a MH diagnosis and those receiving care. Veterans with TBI histories were more likely to have the opportunity to participate in a full course of psychotherapy [40,41] as they were more likely to attend at least eight psychotherapy visits. However, we do not know if those sessions were with a single provider nor do we have data regarding session content, so it is unclear if veterans completing at least eight sessions actually completed a full course of an evidence-based psychotherapy. We do know that veterans who attended less than eight sessions did not complete enough visits for an adequate dose of an evidence-based psychotherapy [40,41].

These results lead to clinical implications and hypotheses for future research. Clinically, it is important to be mindful of the index diagnoses in addition to MH comorbidities, such as personality and substance use disorders, as they are also related to greater service utilization. Veterans may also be more comfortable seeking TBI-related services than MH services as there is less stigma associated with medical than MH diagnoses. Regardless of TBI history, veterans with psychiatric diagnoses should be educated about the effective anxiety, depression, and PTSD treatments available at all VHA medical centers. Providers can correct any misunderstandings that a veteran may have about how a TBI history will impact his/her ability to benefit from a course of psychotherapy because, fortunately, veterans with and without TBI histories see symptom reduction with treatment [15,34]. In terms of research, next steps include examining what type of psychotherapies and medications returning veterans are receiving, and whether these treatments are focusing on TBI in addition to the mental health symptoms.

### Limitations and strengths

A limitation of using national VHA databases for diagnostic information is that there is no independent verification of diagnostic validity through structured clinical interviews or assessments. Similarly, under recognition of TBI in the VHA may be a limitation. Although it is a national mandate that all veterans be screened for TBI, only 92% of veterans have a documented TBI screen [9]. We collapsed TBIs of all severity levels as ICD-9 codes do not reliability distinguish between TBI severity levels [39], and the data did not allow us to distinguish between combat and civilian TBI. Parceling apart these specifics may be useful for future research. We identified OEF/OIF veterans by their birthdates and combat experiences rather than a formal list, and this method may have excluded older veterans born before 1973 who served in OEF/OIF and another war era. We do not know how many veterans died during the one year follow up period, which may skew the service utilization data. Also, we cannot determine whether the MH specialty services were provided in polytrauma clinics or traditional MH specialty clinics, which would be helpful in determining which providers are taking the lead in delivering MH services to veterans with psychiatric diagnoses and TBI histories. Finally, examining the types of medications prescribed to veterans with TBI histories and MH disorders, and if the veterans are compliment with these medications are important questions for future studies.

Strengths of this study include its use of a national subsample of OEF/OIF combat veterans, affording us abundant statistical power with a large representative sample and results that will likely generalize to other OEF/OIF veterans with PTSD, anxiety, and depression with and without TBI. Understanding the unique service utilization trends of this younger generation of veterans is imperative, as they will likely differ from those of previous veteran generations as the nature of their injuries and quantity and content of deployments are vastly different than other war eras [12]. Having access to demographics, index diagnoses, and comorbidities allowed us to examine all diagnoses in one regression model and control for other variables to examine each predictor’s unique contribution while holding other predictors constant.

## Conclusion

Among a national subsample of veterans with psychiatric diagnoses, those with TBI histories attended more psychotherapy and medication management visits than those without TBI histories. Veterans with TBI histories were more likely than veterans without TBI histories to receive eight sessions of psychotherapy, which provides the opportunity to participate in a full course of psychotherapy. Future research should examine if the increase in services used by veterans with TBI histories is resulting in reduction of MH symptoms and overall recovery for these veterans.

## Abbreviations

ICD-9: Classification of Disorders - 9^th^ Edition
MH: mental health
mTBI: mild-severity traumatic brain injury
OEF/OIF/OND: Operation Enduring Freedom/Operation Iraqi Freedom/Operation New Dawn
PTSD: posttraumatic stress disorder
TBI: traumatic brain injury
VHA: Veterans Health Administration
VIRec: Veterans Affairs Information Resource Center
